# Successful TB treatment induces B-cells expressing FASL and IL5RA mRNA

**DOI:** 10.18632/oncotarget.12184

**Published:** 2016-09-22

**Authors:** Ilana C. van Rensburg, Chandre Wagman, Kim Stanley, Caroline Beltran, Katharina Ronacher, Gerhard Walzl, Andre G. Loxton

**Affiliations:** ^1^ SA MRC Centre for TB Research, DST/NRF Centre of Excellence for Biomedical Tuberculosis Research, Division of Molecular Biology and Human Genetics, Faculty of Medicine and Health Sciences, Stellenbosch University, Cape Town, South Africa

**Keywords:** FasL, IL5Ra, B-cells, regulatory cells, TB treatment, Immunology and Microbiology Section, Immune response, Immunity

## Abstract

Activated B-cells increase T-cell behaviour during autoimmune disease and other infections by means of cytokine production and antigen-presentation. Functional studies in experimental autoimmune encephalomyelitis (EAE) indicate that B-cell deficiencies, and a lack of *IL10* and *IL35* leads to a poor prognosis. We hypothesised that B-cells play a role during tuberculosis. We evaluated B-cell mRNA expression using real-time PCR from healthy community controls, individuals with other lung diseases and newly diagnosed untreated pulmonary TB patients at three different time points (diagnosis, month 2 and 6 of treatment).

We show that *FASLG, IL5RA, CD38* and *IL4* expression was lower in B-cells from TB cases compared to healthy controls. The changes in expression levels of *CD38* may be due to a reduced activation of B-cells from TB cases at diagnosis. By month 2 of treatment, there was a significant increase in the expression of *APRIL* and *IL5RA* in TB cases. Furthermore, after 6 months of treatment, *APRIL, FASLG*, *IL5RA and CD19* were upregulated in B-cells from TB cases. The increase in the expression of *APRIL* and *CD19* suggests that there may be restored activation of B-cells following anti-TB treatment. The upregulation of *FASLG* and *IL5RA* indicates that B-cells expressing regulatory genes may play an important role in the protective immunity against *M.tb* infection. Our results show that increased activation of B-cells is present following successful TB treatment, and that the expression of *FASLG* and *IL5RA* could potentially be utilised as a signature to monitor treatment response.

## INTRODUCTION

T-cell driven cell-mediated immunity, has been the focus of studies investigating host immunity against *M.tb* infection [1]-[3] and TB disease. However, an increasing number of studies are indicating that the role of B-cells in the protective immunity against *M.tb* infection has been underestimated. B-cells are the key players during humoral immunity and produce antibodies in response to invading pathogens. Studies found that humoral immunity may enhance protection against *M.tb* infection [4]-[6]. Furthermore, B-cells have additional functions that may be essential for host protection during infection and disease. Activated B-cells change T-cell behaviour during autoimmune disease and other pathogenic infections by means of cytokine production and antigen-presentation. Effector B-cells can either produce Interferon (IFN)-γ and Interleukin (IL)-12 (Be-1 cells) or IL-2, IL-13 and IL-4 (Be-2 cells) depending on whether the cells are primed by Th1 or Th2 cells, respectively [7], [8]. These cytokine-producing effector B-cells are able to amplify T-cell responses in a cytokine-dependent manner [9], [10].

B-cells also display regulatory phenotypes, which secrete IL-10, IL-35, and express FAS ligand (FasL). Functional studies in EAE indicate that B-cell deficiencies, and a lack of *IL10* and *IL35* lead to a poor prognosis[11]. B-cells limited EAE pathogenesis by means of IL-35 secretion, which decreased the accumulation of pathogenic cells in the target organ. FasL-expressing B-cells have a similar effect during autoimmune disease. These B-cells induce apoptosis of CD4+ T-cells, have the potential to reduce the inflammatory responses during autoimmune disease and are induced by IL-5 [12]. High levels of FasL expression was observed in B-cells activated via the Toll-Like Receptor (TLR) 9 agonist CpG [13]. We hypothesised that B-cells play a similar role during tuberculosis disease. Transcriptional approaches are useful for the discovery of biomarkers for the diagnosis and measurement of treatment response [14]-[17]. We evaluated patterns in the expression of B-cell genes to better our understanding of B-cell behaviour during *M.tb* infection and tuberculosis (TB). Real-time PCR was utilised to assess the expression of gene transcripts of cytokines, together with genes involved in B-cell activation and effector functions.

## RESULTS

### Comparison of gene expression between TB, OLD and Control groups

In order to evaluate the differences in the expression of the 12 specific genes in purified B-cells between healthy controls (Ctrl), TB cases and individuals with other lung diseases (OLD), the delta Ct method was utilised. All analyses were carried out by means of the online software provided by SABiosciences.

**Figure 1:**
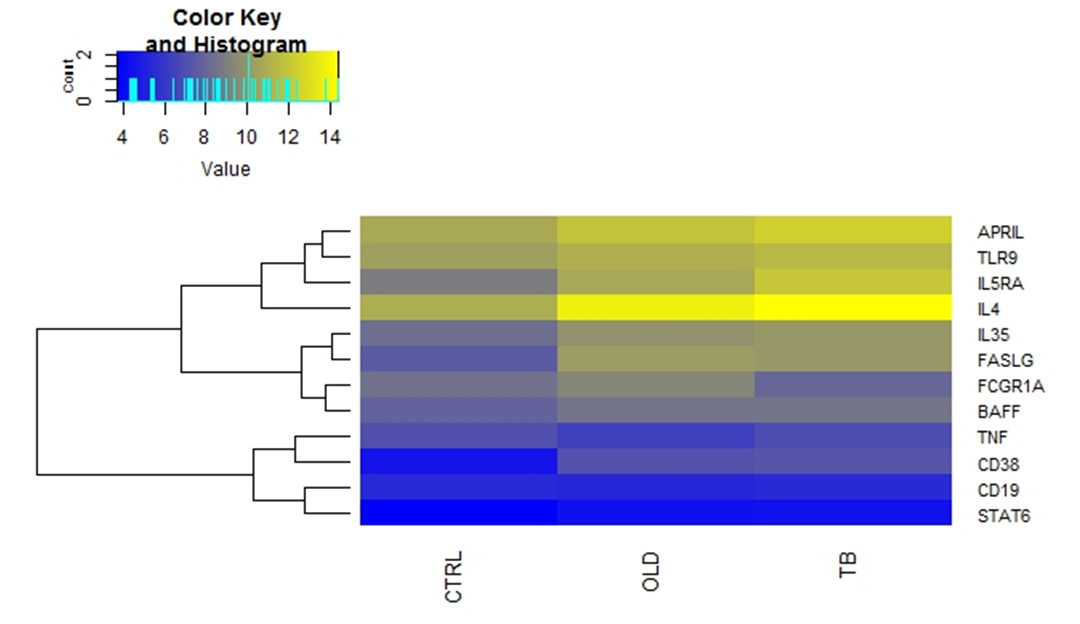
Differences in mRNA expression between TB and Control groups Comparison of the average gene expression between the respective groups. Where Ctrl are healthy controls, OLD are individuals with other lung diseases and TB are individuals is TB disease. Heatmap, which was generated using R statistical packages, depicting changes in normalized expression values (dCt). Genes with low expression levels are depicted in yellow, whereas genes with high expression levels are depicted in blue.

The expression of *APRIL, BAFF, CD19, IL4, FCGR1A, IL35, IL5RA, TNF and TLR9* was similar between Ctrl and OLD groups (*p* > 0.05) (Table 2). In contrast, *FASLG* and *CD38* expression was significantly decreased in OLD compared to Ctrl (*p* < 0.01; Table 2). There were no differences in the expression of all the genes between individuals with other lung diseases and TB cases*.* When comparing the expression of the genes between healthy controls and TB cases, we found that there was a significant decrease in the expression of *IL4*, *FASLG, IL5RA* and *CD38* (*p* < 0.05) (Table 2). The relative gene expression changes between the CTRL, OLD and TB groups are shown in the form of a heat map (Figure 1), which demonstrates differential expression of genes between the control group and the TB group is evident.

**Table 1:** Clinical and demographic characteristics of study participants

	TB	CTRL	OLD
No. of Female	8	10	5
No. of Male	12	0	5
QuantiFERON status (Dx)	NA	POSITIVE	5 POSITIVE
			5 NEGATIVE
Sputum-culture status (Dx)	POSITIVE	NEGATIVE	NEGATIVE

**Table 2:** Gene expression differences between TB and Control Groups

GENES	Ctrl versus OLD		Ctrl versus TB		OLD versus TB	
	Log2 Fold Change	*p*-value	Log2 Fold Change	*p*-value	Log2 Fold Change	*p*-value
***APRIL***	−2.08	*p* > 0.05	−3.03	*p* > 0.05	−1.46	*p* > 0.05
***TLR9***	−1.60	*p* > 0.05	−2.10	*p* > 0.05	−1.31	*p* > 0.05
***IL5RA***	−3.54	*p* > 0.05	**-8.26**	*p*** < 0.01**	−2.33	*p* > 0.05
***IL4***	−7.03	*p* > 0.05	**-10.70**	*p*** < 0.01**	−1.52	*p* > 0.05
***IL35***	−2.77	*p* > 0.05	−3.35	*p* > 0.05	−1.21	*p* > 0.05
***FASLG***	**-6.26**	*p*** < 0.01**	**-5.70**	*p*** < 0.01**	1.10	*p* > 0.05
***FCGR1A***	−1.78	*p* > 0.05	1.39	*p* > 0.05	2.47	*p* > 0.05
***BAFF***	−1.71	*p* > 0.05	−1.74	*p* > 0.05	−1.02	*p* > 0.05
***TNF***	**1.63**	*p*** < 0.05**	1.12	*p* > 0.05	−1.45	*p* > 0.05
***CD38***	**-6.06**	*p*** < 0.01**	**-6.59**	*p*** < 0.01**	−1.09	*p* > 0.05
***CD19***	1.08	*p* > 0.05	−1.03	*p* > 0.05	−1.12	*p* > 0.05
***STAT6***	−1.57	*p* > 0.05	−1.66	*p* > 0.05	−1.06	*p* > 0.05

The fold change values represent the changes in gene expression between the respective groups, as calculated by SABiosciences online software. Positive values indicate an increase in expression, (negative value indicates a decrease in expression) between groups. Kruskal-Wallis tests were used for comparisons, and a *p*-value < 0.05 is considered significant and is highlighted in bold.

### Differential gene expression during Anti-TB treatment

The expression of specific genes in purified B-cells was compared between diagnosis (before treatment started), month two (the time point frequently used to evaluate sputum culture conversion) and month six (end of treatment) following the initiation of anti-TB treatment. There were no significant differences in the expression of *BAFF, CD19, FASLG, IL4, CD38, FCGR1A, IL35, TNF* and *TLR9* between diagnosis and month 2 of treatment (*p* > 0.05) (Table 3). However, *APRIL* and *IL5RA* were differentially expressed between these two time points with fold changes of 1.85 (*p* < 0.05) and 1.87 (*p* < 0.05) respectively (Table 3).

**Figure 2:**
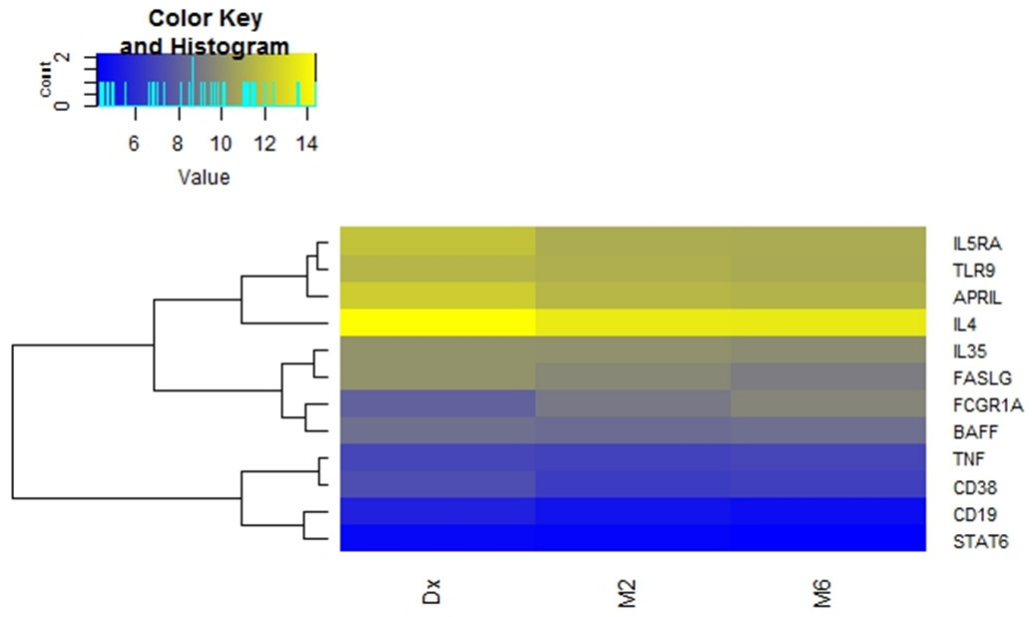
Differential mRNA expression over the course of anti-tuberculosis treatment Heat map depicting changes in normalized expression values (dCt). Genes with low expression levels are depicted in yellow, whereas genes with high expression levels are depicted in blue. **a.** Group comparisons showing gene expression for individual participants. **b.** Comparison of the average gene expression between the respective groups.

**Figure 3:**
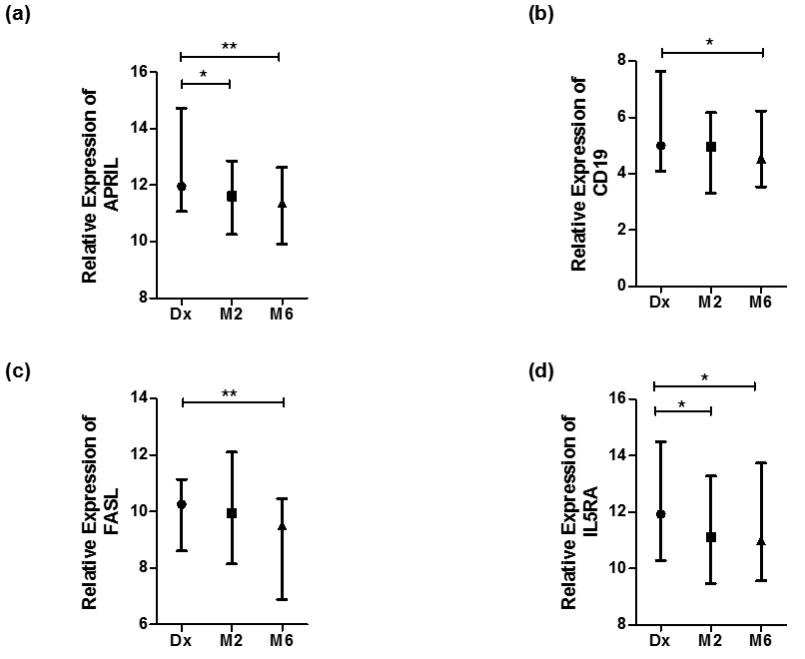
Genes differentially expressed during anti-TB treatment Normalized expression values of genes as calculated using the dCt method (Ct (gene) - Ct (housekeeping gene)). Statistical differences calculated by means of Kruskal Wallis tests, where significant differences are indicated by an asterisk (* = *p* < 0, 05 or ** = *p* < 0.01). Data represented as median dCt with bars representing range. High dCt values indicate low gene expression, and low dCt values indicate high gene expression. **a.** APRIL, **b.** CD19, **c.** FASL and **d.** IL5RA.

When comparing the expression of the 12 genes between diagnosis and month six of treatment, *APRIL, CD19, FASL and IL5RA* were differentially expressed (Table 3 and Figure 3). There was an increase in the expression of all the latter genes at month 6. The expression of *BAFF, STAT6, IL35, TNF, CD38* and *TLR9* was similar at diagnosis and month six of treatment. The relative gene expression is depicted in a heat map for individual samples (Figure 2a) and clustered as groups (Figure 2b).

**Table 3:** Gene expression in TB group over 6 months of treatment

GENES	Dx vs M2		Dx vs M6		M2 vs M6	
	Log2 Fold Change	*p*-value	Log2 Fold Change	*p*-value	Log2 Fold Change	*p*-value
*APRIL*	**1.85**	***p* < 0.05**	**2.06**	***p* < 0.01**	**1.12**	*p* > 0.05
*IL5RA*	**1.87**	***p* < 0.05**	**1.96**	***p* < 0.05**	**1.05**	*p* > 0.05
*TLR9*	1.18	*p* > 0.05	1.35	*p* > 0.05	1.15	*p* > 0.05
*IL4*	1.79	*p* > 0.05	1.84	*p* > 0.05	1.03	*p* > 0.05
*IL35*	1.04	*p* > 0.05	1.21	*p* > 0.05	1.17	*p* > 0.05
*FASLG*	1.33	*p* > 0.05	**1.87**	***p* < 0.01**	1.40	*p* > 0.05
*FCGR1A*	−1.91	*p* > 0.05	−2.74	*p* > 0.05	−1.43	*p* > 0.05
*BAFF*	1.13	*p* > 0.05	1.00	*p* > 0.05	−1.12	*p* > 0.05
*TNF*	1.10	*p* > 0.05	−1.02	*p* > 0.05	−1.12	*p* > 0.05
*CD38*	1.65	*p* > 0.05	1.48	*p* > 0.05	1.11	*p* > 0.05
*CD19*	1.49	*p* > 0.05	**1.77**	***p* < 0.05**	1.18	*p* > 0.05
*STAT6*	1.08	*p* > 0.05	1.21	*p* > 0.05	1.12	*p* > 0.05

The fold change values represent the changes in gene expression between the respective groups, as calculated by SABiosciences online software. Positive values indicate an increase in expression, whereas a negative value indicates a decrease in expression. Kruskal Wallis tests were used for comparisons, and a p-value ≤ 0.05 is considered significant and is highlighted in bold.

## DISCUSSION

B-cells displaying regulatory phenotypes secrete cytokines such as IL-10 and IL-35, and also express FasL. These FasL-expressing B-cells have predominantly been studied in autoimmune diseases and infections [18]. A study conducted in mice showed that IL-35 secreted by B-cells limited EAE pathogenesis by decreasing the accumulation of pathogenic cells in the target organ [11]. Similarly, B-cell-deficiencies in mice have been linked to a decreased control of *Salmonella* infection [19]. In the current study, *FASLG, IL5RA* and *IL4* expression** were decreased in TB cases at diagnosis compared to healthy controls. In contrast, *FASLG* and *IL5RA* expression were increased after 6 months in TB cases. These results suggest that B-cell activity may be restored following successful anti-TB treatment and could play a role in the protective immunity against *M.tb* infection.

CD38 is a membrane-bound protein which is expressed on the surface of B-cells throughout their lifespan [20]. However, the expression of CD38 is highest on B1 B-cells [21]. Crosslinking of the CD38 receptor on mature B-cells leads to proliferation, isotype switching and reduced apoptosis. Additionally, CD38 acts as a co-stimulatory signal to B-cells activated by means of TLR ligands [22], [23]. Here we show a decline in the expression of *CD38* in B-cells from TB participants compared to healthy controls. This points to a reduction in the activation of B-cells during active *M.tb* infection. APRIL, which is a proliferation inducing ligand, is also associated with the activation of B-cells. We found *APRIL* at lower levels in TB participants compared to healthy controls. However, the expression of this gene was restored after 6 months of treatment, in addition to *CD19*. Together these results suggest that there is an increase in B-cell activation throughout treatment in TB cases. This restored B-cell activation may lead to more interactions between B-cells and helper T-cells due to the upregulation of co-stimulatory molecules [23].

Transcriptional analyses are useful for the identification of biomarkers for diagnostics, as well as monitoring treatment response during tuberculosis and other diseases [14]-[17]. In the current study, *FASLG* and *IL5RA* was downregulated in TB cases at diagnosis compared to healthy controls. The expression of the latter genes were upregulated in TB cases at 6 months of treatment. Collectively, this suggests that *FASLG* and *IL5RA* have the potential to be utilised as a biosignature to monitor success or failure of treatment. Further studies in larger cohorts are required to confirm this.

In conclusion, the results of our pilot study suggest that B-cells expressing regulatory genes could be involved during the immune response to *M.tb* infection. Previous studies of regulatory B-cells focussed mostly on autoimmune diseases and helminth infections. Supplementary transcriptional and functional studies are required to confirm the precise role that B cells play during tuberculosis disease and the mechanisms by which the activation/immune induction is achieved.

## MATERIALS AND METHODS

### Participant recruitment and sample collection

This study was carried out according to the guidelines of the ICH-GCP. All participants provided written informed consent for participation in the study, and the use and storage of the samples for biomarker discovery. Ethical approval was obtained from the Health Research Ethics Committee (Ethics number N10/01/013) of Stellenbosch University and the Departments of Health of the Province of the Western Cape and City of Cape Town.

The study participants were recruited from the Ravensmead/Uitsig community in Cape Town Western Cape. Individuals with newly diagnosed, untreated pulmonary TB were enrolled into the study prior to commencement of anti-TB treatment. The inclusion criteria for the ten healthy controls was a positive IGRA. For TB cases we included participants with a chest radiograph with suggestive signs of TB, clinical symptoms of TB including a chronic cough, weight loss and night sweats, as well as a positive sputum-culture and -smear. The exclusion criteria included prior cases of TB or other lung diseases, and a HIV positive status. In addition, ten individuals with a lung disease (other than TB) were enrolled into the study. Half of the other lung disease (OLDs) (Table 1) were IGRA positive. The individuals in the OLD and healthy control (Ctrl) groups did not present any clinical symptoms of *M.tb* infection.

Peripheral blood was collected from the study participants into sodium heparin tubes, as well as sputum samples for smear/culture tests. The individuals with TB were subsequently followed up at month two and month six of treatment, where blood and sputum samples were collected once more to monitor their response to treatment. Thirteen TB cases had converted to culture negative at month 2 of treatment, and all TB cases were cured at the end of treatment which was confirmed by sputum culture tests.

### Sample preparation, mRNA isolation and quality check

On the day of blood collection, peripheral blood mononuclear cells (PBMCs) were isolated using the ficoll/histopaque separation method (GE Health, Piscataway, NJ). Total B-cells were isolated using the MACS bead separation method through positive selection (B cell isolation kit II, Miltenyi, Germany), and stored in RNAlater^®^ (Life Technologies, USA). The cells were stored in liquid nitrogen until batch analysis.

The cells were thawed on ice and spun down before being washed in Phosphate-buffered saline (PBS). Subsequently, mRNA was isolated using the RNEasy^®^ Mini Kit (Qiagen, Germany) according to manufacturer's instructions. RNA purity and quantification was assessed using NanoDrop 2000 spectrophotometer data. More specifically, the 260/ 280 ratio and the 260/ 230 ratio were measured to assess the purity of the isolated RNA. A 260/280 and 260/230 ratio of at least 1.7 and 1.5 respectively, was considered sufficiently pure for further analysis. The RNA samples were stored at -80 ˚C.

### cDNA synthesis and RT-qPCR

The isolated RNA was thawed on ice and diluted to a concentration of 300ug, and subsequently used to synthesise cDNA. The procedure was carried out in a thermal cycler (Life Technologies, USA) using the First Strand Kit (Qiagen, Germany) according to the manufacturer's instructions. The cDNA was then used as template for quantitative PCR on the ABI 7900HT platform. The RT^2^ Profiler Custom Arrays (Qiagen, Germany) were utilised and manufacturer's instructions were followed. The arrays contained primers for the following genes of interest: *IL35* (NM_000882.3), *IL4*(NM_000589.3), Fas-ligand (*FASLG*) (NM000639.2), *IL5RA* receptor alpha (*IL5RA*) (NM_000564.4), cluster of differentiation 38 (*CD38*) (NM_001775.2), *APRIL* (NM_00198622.1), *BAFF* (NM001145645.2), *TNF-a* (NM_000594.3), Fc gamma receptor 1 alpha (*FCGR1A)* (NM_000566.3), Toll-like receptor 9 (*TLR9*) (NM_017442.3), *STAT6* (NM_001178078.1)*,* as well as two housekeeping genes *B2M* (NM_004048) and *GAPDH* (NM_002046). *FASLG, IL5RA* and *IL35* is associated with regulatory B-cells. Furthermore, *APRIL, CD38* and *TLR9* is involved in B-cells activation.

### Data analysis

The gene expression data obtained from the ABI 7900HT was represented as Ct values, which indicated the earliest visible cycle of amplification. These Ct values were converted to fold change values using the Qiagen online software (www.SABiosciences.com/pcrarraydataanalysis.php). Where applicable, differences were calculated using Prism 5 Software and Kruskal-Wallis tests, where *p* < 0.05 indicates a statistically significant difference. Heatmaps were generated using delta Ct (dCt) data and R statistical packages.
